# Good data with ‘bad’ reflections: the employment of non-spherical scattering factors in the redetermination of the structure of *O*-ethyl *N*-phenyl­carbamate

**DOI:** 10.1107/S2053229625005959

**Published:** 2025-07-10

**Authors:** Peter G. Jones

**Affiliations:** a Institut für Anorganische und Analytische Chemie, Technische Universität Braunschweig, Hagenring 30, D-38106 Braunschweig, Germany; The University of Western Australia, Australia

**Keywords:** thio­urea, carbamate, hy­dro­gen bonding, nonspherical scattering factors, crystal structure

## Abstract

The structure of *O*-ethyl *N*-phenyl­thio­carbamate, C_9_H_11_NOS (**2**), has been redetermined, confirming the results obtained in three earlier structure determinations. The higher data quality provided by modern diffractomers has enabled a reliable analysis (absent from the earlier reports) of the hy­dro­gen bonding. However, conventional refinement of the structure of **2** was unsatisfactory because of the large number of extremely badly-fitting reflections, leading to many *checkCIF* ‘ALERT A’ messages that might be detrimental to ease of publication. A refinement using nonspherical scattering factors effectively eliminated this problem. There are three independent mol­ecules of **2** in the asymmetric unit; two are directly connected by two N—H⋯S hy­dro­gen bonds, forming a dimer with the well-known *R*_2_^2^(8) motif. The other mol­ecule forms a topologically identical but inversion-symmetric dimer. Each type of dimer occupies a different region parallel to the *ac* plane (mol­ecule 1, *y* ≃ 0; mol­ecules 2 and 3, *y* ≃  and ). All three molecules lie in planes parallel to (031). The title com­pound is effectively isotypic to 1-ethyl-3-phenyl­thio­urea (another known structure for which the hy­dro­gen bonding was not analysed) because its EtNH group, like the EtO group of **2**, is not involved in hy­dro­gen bonding.

## Abstract 2

The structure of *O*-ethyl *N*-phenyl­thio­carbamate has been redetermined. Layers of hy­dro­gen-bonded dimers lie parallel to (031). A refinement using nonspherical scattering factors greatly reduced the number and severity of badly-fitting reflections.

## Introduction

In a recent investigation of well-formed crystals that were believed to be an organic thiol, containing the elements C, H, N and S, preliminary diffractometer investigations (using the routine ‘What is this?’; Rigaku OD, 2024 ▸) suggested that the com­pound was in fact 1-ethyl-3-phenyl­thio­urea (**1**), the structure of which is known [Singh *et al.*, 2015 ▸; room-tem­per­a­ture data; Cambridge Structural Database (CSD, Version 5.46 of November 2024; Groom *et al.*, 2016 ▸) refcode NOQTUK]. Because this publication did not give a detailed account of the hy­dro­gen bonding, and in view of my inter­est in structures of ureas and thio­ureas and their adducts (Strey & Jones, 2018 ▸, and references therein), it was decided to measure a new dataset for this structure. Accordingly, high-quality data were col­lec­ted to a resolution limit of 0.45 Å at 100 K.

During the data collection, it became clear (using the routine ‘Autochem’) that one of the two supposed HN–ethyl groups was in fact O–ethyl, so that the com­pound was *O*-ethyl *N*-phenyl­thio­carbamate (**2**); the ‘What is this?’ routine had only been given the elements C, H, N and S and so could not assign the O atom. This structure too is known, with three database entries of the refcode family PINPIL: Taylor & Tiekink (1994 ▸), room-tem­per­a­ture data; Nieger *et al.* (2019 ▸), deposited data measured at 123 K; and Alsayari *et al.* (2021 ▸), room-tem­per­a­ture data with no reference to the earlier structures. It is notable that the unit-cell constants of both com­pounds are very similar (Table 1 ▸), and both have *Z*′ = 3, but this does not seem to have been commented on. The CIF of Nieger *et al.* (2019 ▸) included three N—H⋯S hy­dro­gen bonds; the other two publications did not discuss these.

In the present article, I discuss the hy­dro­gen bonding of **2** and com­pare it to that of **1**. I also describe problems with the conventional refinement of this and other high-resolution datasets (namely that residual electron density can be high, and there can be many ‘bad’ reflections with large differences between calculated and observed structure factors) and the methods used to overcome these problems.
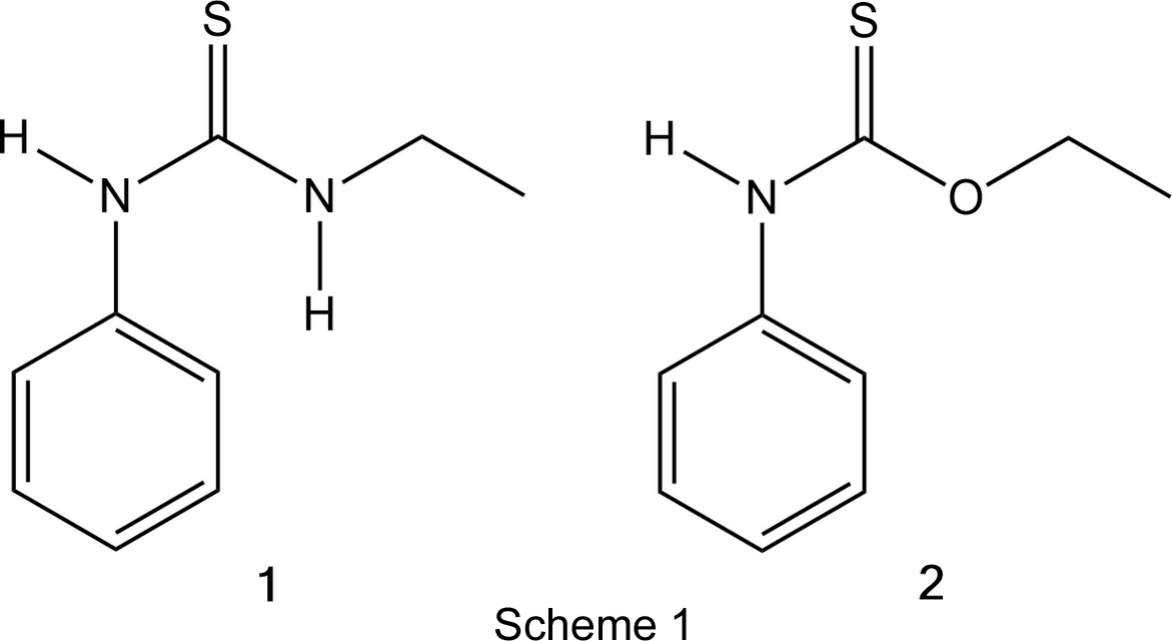


## Experimental

### Synthesis and crystallization

Crystals arose by chance from an experiment designed to deliver an organic thiol. Clearly, the reaction did not proceed as expected; there may have been decom­position by accidental access of atmospheric moisture.

### Refinement

Details of the measurements (necessarily identical for both refinements of the same data!) and refinements are given in Table 2 ▸. The standard refinement (column ‘**2**_IAM’) employed *SHELXL* (Sheldrick, 2015*b* ▸). H atoms of NH groups were refined freely. Methyl groups were refined as idealized rigid groups, with C—H = 0.98 Å and H—C—H = 109.5°, and allowed to rotate but not tip (command ‘AFIX 137’). Other H atoms were included using a riding model, starting from calculated positions, with C—H = 0.95 Å for aromatic and 0.99 Å for methyl­ene H atoms. For the *NoSpherA2* refinement (column ‘**2**_NoSpherA2’ in Table 2 ▸), the wavefunction was calculated using *ORCA* (Neese *et al.*, 2020 ▸; Neese, 2022 ▸), using the B3LYP hybrid functional and the def2-SVP basis set (see also the following section).

## Results and discussion

### Structural commentary

**Figure 1:**
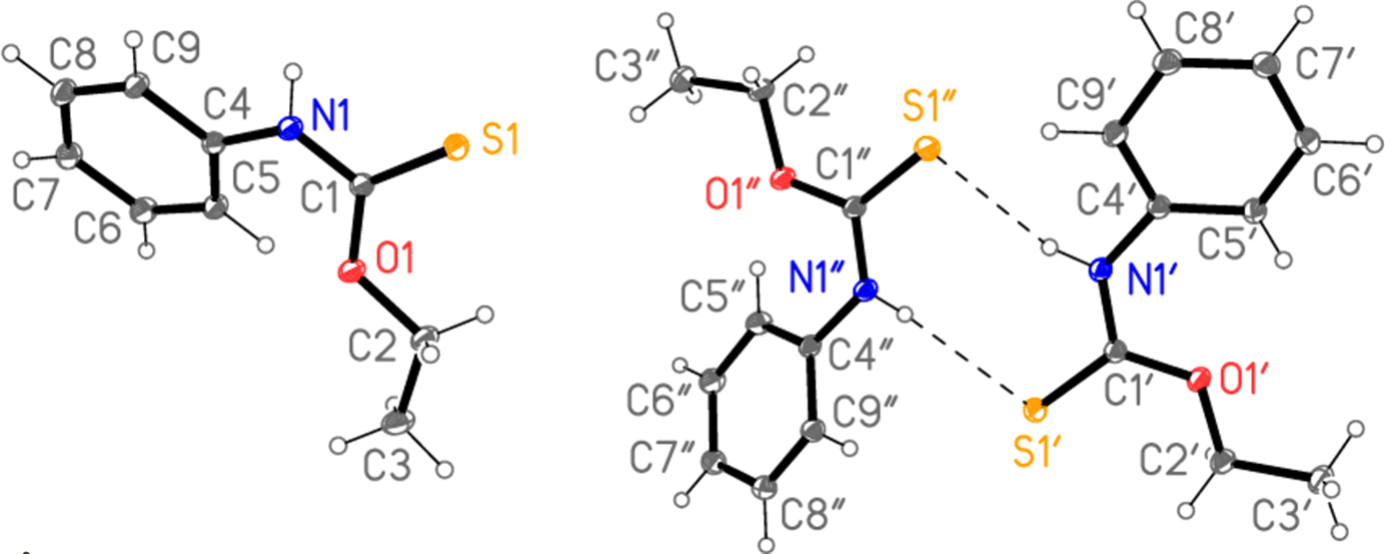
The asymmetric unit of com­pound **2** in the crystal. Ellipsoids correspond to the 50% probability level. The dashed lines indicate hy­dro­gen bonds.

The structure of **2** is shown in Fig. 1 ▸, with selected mol­ecular dimensions in Table 3 ▸. All values discussed here are those from the standard refinement (labelled as ‘**2**_IAM’ in the Tables; for ‘**2**_NoSpherA2’, see Section 4, *Use of non-spherical scattering factors*). As established by Taylor & Tiekink (1994 ▸), the asymmetric unit of **2** contains three independent but closely similar mol­ecules; the second and third mol­ecules are distinguished here by atom numbers with primes (′) or double primes (′′), respectively. A suitable choice of the asymmetric unit shows that mol­ecules 2 and 3 are connected by two N—H⋯S hy­dro­gen bonds, involving a ring of graph set 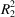
(8).

As discussed by Taylor & Tiekink (1994 ▸), bond lengths and atoms may be considered normal. The atom sequence C4—N1—C1—S1 is anti­periplanar, whereas C2—O1—C1—S1 is synperiplanar (see torsion angles in Table 3 ▸). The sequence H01—N1—C1—S1 is then necessarily synperiplanar, which facilitates the observed hy­dro­gen-bonding pattern. The central group of atoms C1, C2, C4, N1, O1 and S1 is essentially planar, with an r.m.s. deviation of 0.033 Å; atom C3, the terminal C atom of the ethyl group, lies outside this plane by only 0.126 (1) Å. The inter­planar angle to the arene ring is 28.73 (1)°. The corresponding values for mol­ecules 2 and 3 are: r.m.s. deviations 0.034 (1) and 0.021 (1), C3 deviations 0.108 (1) and 0.108 (1) Å, and inter­planar angles 11.94 (2) and 31.31 (1)°, respectively. The inter­planar angle thus varies somewhat between the three mol­ecules. The close intra­molecular contacts H5⋯O1, included in the list of hy­dro­gen bonds (Table 4 ▸), are associated with the small inter­planar angles.

### Supra­molecular features

**Figure 2:**
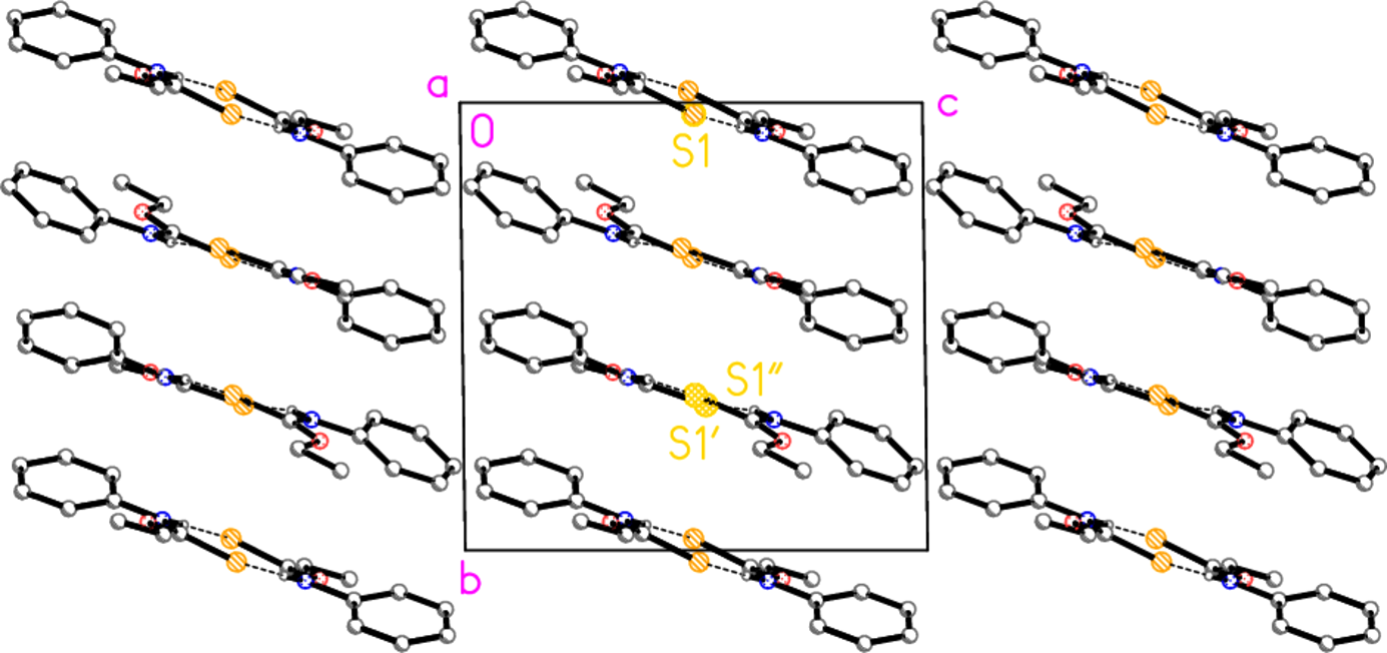
Packing of com­pound **2** projected parallel to the *a* axis. The layers are shown edge-on. Atom labels distinguish the S atoms of the three independent mol­ecules. For all packing diagrams, the dashed lines indicate hy­dro­gen bonds; H atoms not involved in hy­dro­gen bonding have been omitted for clarity.

**Figure 3:**
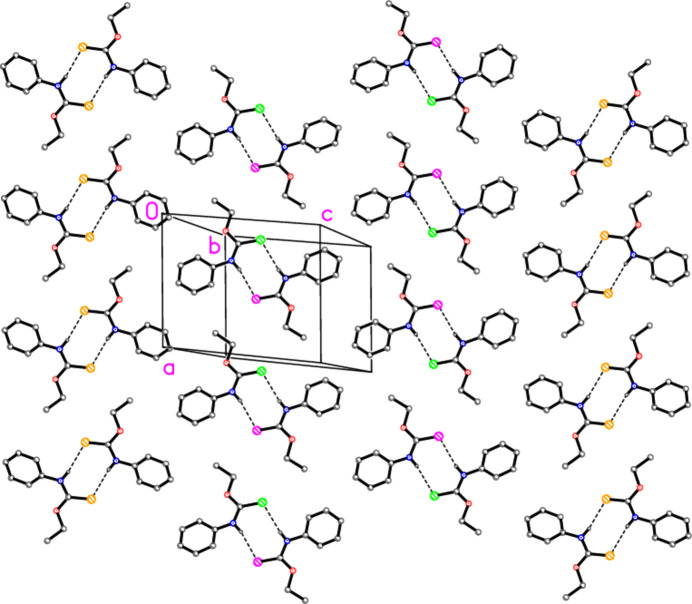
The packing of com­pound **2**, showing a layer viewed perpendicular to (0
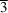
1). S atoms are labelled with different colours: S1 yellow, S1′ green and S1′′ purple.

Hydrogen bond details are given in Table 4 ▸. The packing of com­pound **2** consists of layers of mol­ecules, parallel to (0
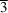
1), connected by N—H⋯S hy­dro­gen bonds to form dimers with the well-known 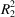
(8) motif; these layers are seen edge-on in Fig. 2 ▸, running diagonally from top left to bottom right. One type of dimer involves mol­ecule 1 only, which forms hy­dro­gen bonds *via* inversion symmetry; these mol­ecules lie horizontally, top and bottom in Fig. 2 ▸, occupying the regions *y* ≃ 0 and 1. Mol­ecules 2 and 3 assemble in an exactly equivalent manner, but without crystallographic symmetry of the dimers, and occupy the two horizontal regions in the centre of Fig. 2 ▸, at *y* ≃ 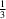 and 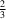. A layer com­prising all three mol­ecules is shown in Fig. 3 ▸. One translation between translationally equivalent dimers is [100]; the other, lateral, translation may be chosen parallel to [013], confirming the plane (*via* the zone law) as (0
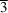
1). The values ‘3’ in the cited planes and vectors are clearly directly connected with the *Z*′ value of 3.

The contacts H9⋯S1 across the dimers may be regarded as ‘weak’ hy­dro­gen bonds that may provide additional stabilization, although they are not drawn explicitly in the figures. It is notable that the O atoms are not involved in hy­dro­gen bonding; C—O—C moieties are generally considered as less probable hy­dro­gen-bond acceptors (Allen *et al.*, 1999 ▸).

There is no π–π stacking and the shortest C—H⋯*Cg* distance is 2.85 Å for H8⋯*Cg*(C4′′–C9′′)(*x* − 1, *y* − 1, *z*) (*Cg* indicates a ring centroid).

**Figure 4:**
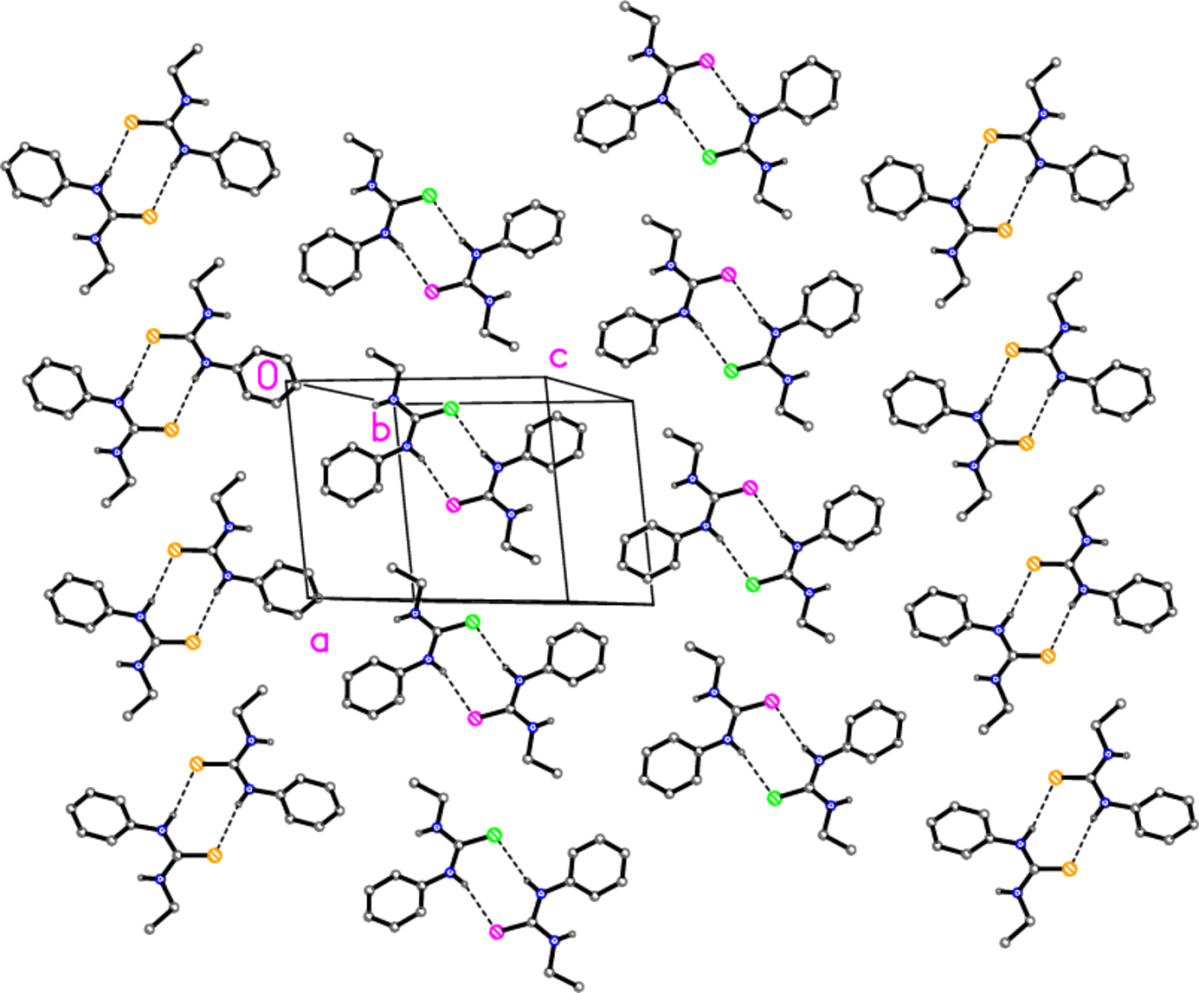
The packing of com­pound **1**, showing a layer viewed perpendicular to (0
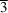
1). As for Fig. 3 ▸, the S atoms of different independent mol­ecules are given different colours. The diagram was drawn from coordinates retrieved from the CSD.

A com­parison of the hy­dro­gen-bonding patterns in com­pounds **1** and **2** shows that they are exactly equivalent, so that the com­pounds may be regarded as isotypic, whereby the O atom of **2** corresponds to one N—H group of **1**; Fig. 4 ▸ shows the layer structure of com­pound **1**. This EtNH group of **1** is thus, perhaps surprisingly, not involved in hy­dro­gen bonding.

### Database survey

The search employed the routine *ConQuest* (Bruno *et al.*, 2002 ▸), as implemented in the Cambridge Structural Database (Version 5.46 of November 2024; Groom *et al.*, 2016 ▸). It was designed to find only structures containing **1** or **2**. In addition to the stuctures of **2** alone, it also found the 2:1 adducts (cocrystals) of **2** with 4,4′-bi­pyridine (refcode MEWJIK; Yeo & Tiekink, 2018 ▸) and *trans*-1,2-bis­(pyridin-4-yl)ethene (UHOSEQ; Ellis *et al.*, 2009 ▸).

## Use of non-spherical scattering factors

Modern diffractometers, with their powerful X-ray sources, highly sensitive detectors and reliable low-tem­per­a­ture attachments, can nowadays deliver data of a quality that I could not have dreamt of when I began to employ X-ray structure determination half a century ago (Jones *et al.*, 1975 ▸; Abu-Zaied *et al.*, 2024 ▸). Even for small crystals of organic com­pounds, data of significant intensity can often be recorded to 2θ 70° or more (for Mo *K*α radiation); inspection of the data reduction shows that such data are often present even if they are too faint to be recognised on diffractometer screen images. One slightly disturbing aspect in my recent experience has been the tendency for refinements of such datasets to give rise to appreciable numbers of badly fitting reflections; these are listed by *SHELXL* (Sheldrick, 2015*b* ▸) as the ‘Most Dis­agree­able Reflections’. Thus, in a recent structure (C_19_H_13_ClN_4_OS), measured to 90° (Metwally *et al.*, 2025 ▸), there were 13 reflections with deviations between 7σ and 10.2σ. Omitting the worst five from the refinement did not improve the *wR*2 value, so they were retained. Often, however, omitting a handful of ‘bad’ reflections improves the refinement somewhat, and this is the strategy I have often employed, even if the OMIT command is rather a blunt instrument. Similarly, a recent (unpublished) structure (C_19_H_19_N_2_O_3_P), measured to 105°, had 28 reflections with deviations between 7σ and 13σ.

The new dataset for **2** proved to be an extreme case of this infelicity; in a long list of ‘disagreeable reflections’, the 10 worst-fitting reflections (Table 5 ▸) had deviations as high as 10.3–18.6σ. All these reflections have *F*_o_^2^ >> *F*_c_^2^, are quite weak [the highest *F_c_*/*F_c_*(max) is 0.026] and occur at moderate resolution (1.1–2.0 Å). This effect presents a significant challenge for accurate refinement, and might well prevent the structure being published, if editors and/or referees inter­preted the corresponding *checkCIF* ‘ALERT A’ messages strictly. A pragmatist might decide not to collect data to such high angles (or to use a resolution cutoff during refinement), in order to avoid the problem; cutting the data at the ‘IUCr limit’ of 0.84 Å reduces the error/e.s.d. values dramatically (Table 5 ▸). I wish to stress that I do not recommend doing this, but one can see the temptation!

For **2**, there were two possible explanations for this effect. Some powder rings, probably attributable to a slightly mis­moun­ted loop, might have given rise to erroneous intensities; however, assiduous removal of the affected frames did not improve the refinement. Alternatively, the use of spherical atom scattering factors might be questioned.

It is well known that the use of spherical atom scattering factors (the independent atom model, IAM) is not ideal, because the electron distribution in any real crystal must involve valence electrons, and thus cannot correspond exactly to spherically symmetric atoms. Consistent with this, IAM refinements using high-angle data generally lead to significant residual electron density with maxima at the mid-point of covalent bonds; this can lead to checkCIF ‘C alerts’ of type PLAT094 (*Ratio of maximum/minimum residual density*) and ‘G alerts’ of type PLAT978 (*Number C—C bonds with positive residual density*), although the latter are probably intended as a check against fraudulent data. Nevertheless, in practice, and in the absence of a better procedure that can be simply applied, spherical scattering factors continue to be used; any errors thus arising are considered to be small and tolerable.

Recent attempts to use non-spherical scattering factors include the program *NoSpherA2* (Kleemiss *et al.*, 2021 ▸, and references therein). This operates under the *OLEX2* platform (Dolomanov *et al.*, 2009 ▸; Bourhis *et al.*, 2015 ▸), which normally offers the alternatives of *SHELXL* (Sheldrick, 2015*b* ▸) or *olex2.refine* for structure refinement; however, only the latter is suitable for *NoSpherA2*. The wavefunction of the mol­ecule is calculated and used to determine the scattering factors for each atom, which are then employed in the subsequent refinement. One iteration of the procedure is generally sufficient to provide suitable scattering factors; subsequent refinement cycles omit wavefunction calculations and thus are much faster. Some aspects of the procedure are at first sight somewhat disconcerting, perhaps because they are unfamiliar to the inexperienced user: it might be regarded as somewhat circular (using the structure to determine scattering factors, then using these to refine the structure); for organic structures, H atoms can often be refined freely and anisotropically; refinement times are (on my aged com­puter) typically around 15 minutes for a full calculation including wavefunction, instead of a few seconds; *R* values are very low, and standard uncertainties of derived parameters are also very low; total file sizes for the refinement, excluding frames, can be in the GB rather than MB range; and the user is forced to use *OLEX2* because (to the best of my knowledge) the method is not readily available in other refinement programs/platforms. A clear advantage is that bond lengths involving H atoms tend towards the ‘correct’ values rather than the artificially shortened values from traditional refinement. Hill & Boeré (2025 ▸, and references therein) have published a valuable and thought-provoking review detailing their extensive experiences with *NoSpherA2*; they mention many advantages of its use and argue forcefully that it should become the standard refinement method. They also point to several disadvantages of standard refinement, but do not refer explicitly to problems with ‘bad’ reflections.

**Figure 5:**
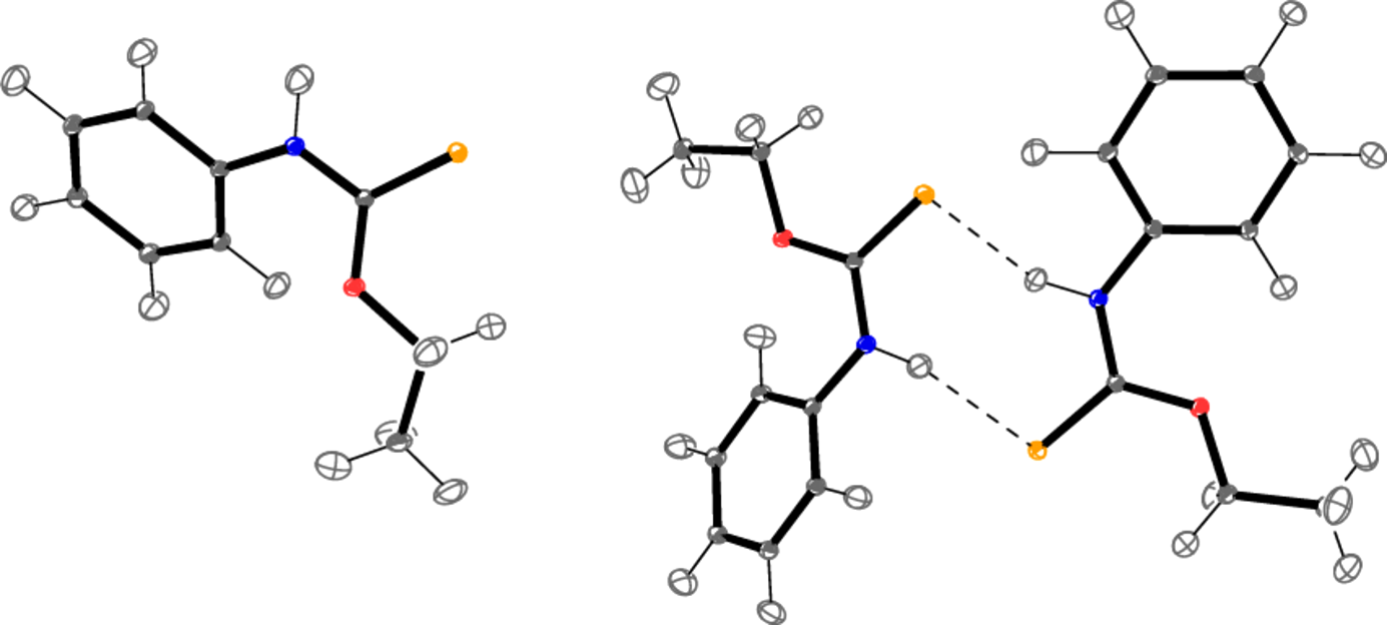
The structure of com­pound **2** after aspherical refinement. Ellipsoids correspond to the 30% probability level, in order to show the H-atom ellipsoids on a reasonable scale. The dashed lines indicate hy­dro­gen bonds. See Fig. 1 ▸ for the atom numbering.

The use of *NoSpherA2* to refine the structure of **2** led to a great improvement, in that all previously ‘bad’ reflections now fitted well (none of the ten previously worst reflections had an absolute deviation of more than 4σ; for all reflections, the highest deviation was 6.7σ and all others were < 4.8σ). The ellipsoid plot (Fig. 5 ▸) is drawn at the 30% probability level to enable inclusion of the anisotropic H atoms. Tables 6 ▸ and 7 ▸ show the results of the *NoSpherA2* refinement; the dimensions of the hy­dro­gen bonds (Table 7 ▸) should be more realistic than those of the standard refinement, in which the bonds to hy­dro­gen are systematically shortened (a disadvantage of standard refinement that crystallographers have come to accept and, largely, ignore).

For the structure C_19_H_13_ClN_4_OS mentioned above, a *NoSpherA2* refinement again removed the worst reflections, so that only one reflection with a deviation >7σ remained. Subjectively, this is a less dramatic improvement than for **2**, so the standard refinement was retained. Similarly, for the structure C_19_H_19_N_2_O_3_P, no reflections with deviations >7σ remained after a *NoSpherA2* refinement. This seems to be a general effect.

Crystallographers, authors, referees and journal editors must decide to what extent the use of programs such as *NoSpherA2* is justified in preference to IAM refinement (perhaps only in extreme cases?), and how the results thus obtained should be com­pared to those of conventional refinement. The need for a decision is implied in the title of the article by Hill & Boeré (2025 ▸): ‘*Small mol­ecule X-ray crystal structures at a crossroads*’. My opinion is that, if a crystal diffracts to 100°, data should be measured to 100°, even if the outlier reflections become more obvious with increasing data quality (judged by the usual criteria, such as 2θ_max_, *R*_int_ and *R*_sigma_); if non-spherical scattering factors then have to be employed for the refinement (because otherwise the ‘bad’ reflections become unpleasantly numerous) so be it. For less well-scattering crystals, the crossroad junction turning to *NoSpherA2* may well, to resort to a mixed metaphor, be a red herring.

## Supplementary Material

Crystal structure: contains datablock(s) 2_IAM, global, 2_NoSpherA2. DOI: 10.1107/S2053229625005959/oc3026sup1.cif

Structure factors: contains datablock(s) 2_IAM. DOI: 10.1107/S2053229625005959/oc30262_IAMsup3.hkl

Structure factors: contains datablock(s) 2_NoSpherA2. DOI: 10.1107/S2053229625005959/oc30262_NoSpherA2sup4.hkl

CCDC references: 2469611, 2469612

## Figures and Tables

**Table 1:** Structure determinations of **1** and **2** and their unit-cell constants For both determinations, the space group is *P*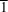 and *Z* = 6.

Compound/refcode	Reference	*T* (K)	*a* (Å)	*b* (Å)	*c* (Å)	α (°)	β (°)	γ (°)	*V* (Å^3^)
**1**/NOQTUK	Singh *et al.* (2015 ▸)	293	9.7037 (8)	12.0974 (10)	12.2300 (10)	89.337 (7)	84.504 (7)	85.224 (7)	1424.1 (2)
**2**/PINPIL	Taylor & Tiekink (1994 ▸)	295	11.972 (4)	12.114 (1)	9.607 (2)	95.52 (1)	94.80 (3)	89.34 (2)	1382.0
**2**/PINPIL01	Nieger *et al.* (2019 ▸)	123	9.6664 (3)	11.7827 (3)	12.1319 (3)	88.829 (1)	84.823 (1)	84.368 (1)	1369.41 (6)
**2**/PINPIL02*^*a*^*	Alsayari *et al.* (2021 ▸)	293	9.6587 (4)	11.7585 (5)	12.1212 (5)	88.807 (2)	84.858 (2)	84.314 (2)	1364.24 (10)
**2**	This work	100	9.6661 (2)	11.7465 (3)	12.1224 (2)	88.8230 (18)	84.8866 (16)	84.2903 (18)	1364.03 (5)

Note: (*a*) the unit-cell constants for PINPIL02 and title structure **2** are closely similar, despite the recorded tem­per­a­ture of PINPIL02 being given as 293 K in the CSD.

**Table 2:** Experimental details For both refinements: C_9_H_11_NOS, triclinic, *P*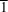, *Z* = 6. The experiment was carried out at 100 K with Mo *K*α radiation using a Rigaku XtaLAB Synergy diffractometer. Absorption was corrected for by multi-scan methods (*CrysAlis PRO*; Rigaku OD, 2024 ▸).

	**2**_IAM	**2**_NoSpherA2
Crystal data		
*M*_r_	181.25	
*a*, *b*, *c* (Å)	9.6661 (2), 11.7465 (3), 12.1224 (2)	
α, β, γ (°)	88.8230 (18), 84.8866 (16), 84.2903 (18)	
*V* (Å^3^)	1364.03 (5)	
μ (mm^−1^)	0.31	
Crystal size (mm)	0.2 × 0.2 × 0.1	

Data collection		
*T*_min_, *T*_max_	0.220, 1.000	
No. of measured, independent and observed reflections [*I* > 2σ(*I*)]	317871, 33252, 23960	
*R*_int_	0.053	
θ values (°)	θ_max_ = 53.9, θ_min_ = 2.1	
(sin θ/λ)_max_ (Å^−1^)	1.136	

Refinement		
*R*[*F*^2^ > 2σ(*F*^2^)], *wR*(*F*^2^), *S*	0.034, 0.108, 1.06	0.024, 0.044, 0.99
No. of reflections	33252	33252
No. of parameters	340	622
H-atom treatment	H atoms treated by a mixture of independent and constrained refinement	All H-atom parameters refined
Δρ_max_, Δρ_min_ (e Å^−3^)	0.63, −0.29	0.38, −0.30

Computer programs: *CrysAlis PRO* (Rigaku OD, 2024 ▸), *SHELXT* (Sheldrick, 2015*a* ▸), *SHELXL2019* (Sheldrick, 2015*b* ▸), *olex2.refine* (Bourhis *et al.*, 2015 ▸), *XP* (Bruker, 1998 ▸) and *publCIF* (Westrip, 2010 ▸).

**Table 3:** Selected geometric parameters (Å, °) for **2**_IAM[Chem scheme1]

S1—C1	1.6702 (4)	N1′—C1′	1.3470 (5)
O1—C1	1.3266 (4)	N1′—C4′	1.4167 (5)
O1—C2	1.4586 (5)	S1′′—C1′′	1.6724 (4)
N1—C1	1.3484 (5)	O1′′—C1′′	1.3241 (5)
N1—C4	1.4191 (5)	O1′′—C2′′	1.4541 (5)
S1′—C1′	1.6727 (4)	N1′′—C1′′	1.3457 (5)
O1′—C1′	1.3274 (5)	N1′′—C4′′	1.4179 (5)
O1′—C2′	1.4604 (5)		
			
C1—O1—C2	118.52 (3)	O1′—C1′—S1′	124.18 (3)
C1—N1—C4	130.50 (3)	N1′—C1′—S1′	121.75 (3)
O1—C1—N1	113.77 (3)	C1′′—O1′′—C2′′	119.55 (3)
O1—C1—S1	124.40 (3)	C1′′—N1′′—C4′′	128.93 (3)
N1—C1—S1	121.82 (3)	O1′′—C1′′—N1′′	112.85 (3)
C1′—O1′—C2′	118.40 (3)	O1′′—C1′′—S1′′	124.79 (3)
C1′—N1′—C4′	131.60 (3)	N1′′—C1′′—S1′′	122.36 (3)
O1′—C1′—N1′	114.04 (3)		
			
C2—O1—C1—N1	−179.41 (4)	C1′—O1′—C2′—C3′	−178.03 (3)
C2—O1—C1—S1	0.53 (5)	C1′—N1′—C4′—C5′	16.53 (7)
C4—N1—C1—O1	−7.65 (6)	C1′—N1′—C4′—C9′	−164.81 (4)
C4—N1—C1—S1	172.42 (3)	C2′′—O1′′—C1′′—N1′′	179.74 (4)
C1—O1—C2—C3	−174.98 (4)	C2′′—O1′′—C1′′—S1′′	−0.49 (6)
C1—N1—C4—C5	−24.14 (6)	C4′′—N1′′—C1′′—O1′′	−4.65 (6)
C1—N1—C4—C9	159.70 (4)	C4′′—N1′′—C1′′—S1′′	175.57 (3)
C2′—O1′—C1′—N1′	−173.55 (3)	C1′′—O1′′—C2′′—C3′′	176.04 (4)
C2′—O1′—C1′—S1′	8.55 (5)	C1′′—N1′′—C4′′—C5′′	36.50 (7)
C4′—N1′—C1′—O1′	−1.46 (6)	C1′′—N1′′—C4′′—C9′′	−146.97 (4)
C4′—N1′—C1′—S1′	176.50 (3)		

**Table 4:** Hydrogen bond geometry (Å, °) for **2**_IAM[Chem scheme1]

*D*—H⋯*A*	*D*—H	H⋯*A*	*D*⋯*A*	*D*—H⋯*A*
N1—H01⋯S1^i^	0.864 (9)	2.533 (10)	3.3867 (3)	169.5 (9)
N1′—H01′⋯S1′′	0.860 (10)	2.538 (10)	3.3766 (4)	165.0 (9)
N1′′—H01′′⋯S1′	0.807 (10)	2.573 (10)	3.3668 (4)	167.7 (10)
C5—H5⋯O1	0.95	2.31	2.8306 (5)	114
C5′—H5′⋯O1′	0.95	2.25	2.8136 (5)	118
C5′′—H5′′⋯O1′′	0.95	2.34	2.7983 (5)	109
C9—H9⋯S1^i^	0.95	3.02	3.7264 (4)	132
C9—H9⋯S1′^ii^	0.95	3.03	3.7860 (4)	138
C9′—H9′⋯S1′′	0.95	2.92	3.6080 (4)	130
C9′′—H9′′⋯S1′	0.95	2.98	3.6864 (4)	132
C2′—H2*A*′⋯S1^iii^	0.99	2.92	3.6862 (4)	135

Symmetry codes: (i) 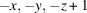; (ii) 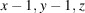; (iii) 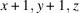.

**Table 5:** The worst ‘disagreeable reflections’ for com­pound **2** (IAM refinement)

*h*	*k*	*l*	Error/e.s.d.	Error/e.s.d. for data cut to 0.84 Å	*F*_c_/*F*_c_(max)	Resolution (Å)
5	1	1	18.62	6.67	0.009	1.92
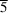	2	4	17.26	5.52	0.006	1.48
3	5	4	14.99	5.80	0.006	1.71
5	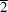	2	13.79	5.33	0.002	1.74
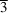	3	9	12.83	3.60	0.001	1.14
5	3	1	11.56	5.78	0.017	1.79
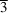	5	0	10.88	3.91	0.009	1.80
5	4	5	10.54	4.37	0.021	1.44
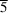	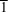	5	10.45	4.90	0.022	1.44
3	5	1	10.33	5.15	0.026	1.98

**Table 6:** Selected geometric parameters (Å, °) for **2**_NoSpherA2

S1—C1	1.6715 (2)	N1′—C1′	1.3471 (3)
O1—C1	1.3239 (3)	N1′—C4′	1.4150 (3)
O1—C2	1.4573 (3)	S1′′—C1′′	1.6746 (2)
N1—C1	1.3481 (3)	O1′′—C1′′	1.3209 (3)
N1—C4	1.4174 (3)	O1′′—C2′′	1.4506 (3)
S1′—C1′	1.6734 (2)	N1′′—C1′′	1.3444 (3)
O1′—C1′	1.3244 (3)	N1′′—C4′′	1.4170 (3)
O1′—C2′	1.4579 (3)		
			
C2—O1—C1	118.704 (17)	N1′—C1′—S1′	121.673 (16)
C4—N1—C1	130.377 (18)	N1′—C1′—O1′	114.246 (18)
O1—C1—S1	124.268 (16)	C2′′—O1′′—C1′′	119.854 (18)
N1—C1—S1	121.763 (15)	C4′′—N1′′—C1′′	128.813 (19)
N1—C1—O1	113.969 (18)	O1′′—C1′′—S1′′	124.602 (16)
C2′—O1′—C1′	118.691 (17)	N1′′—C1′′—S1′′	122.254 (17)
C4′—N1′—C1′	131.471 (19)	N1′′—C1′′—O1′′	113.144 (19)
O1′—C1′—S1′	124.049 (16)		
			
S1—C1—O1—C2	0.52 (2)	C1′—O1′—C2′—C3′	−178.01 (2)
S1—C1—N1—C4	172.539 (16)	C1′—N1′—C4′—C5′	16.53 (3)
O1—C1—N1—C4	−7.51 (3)	C1′—N1′—C4′—C9′	−164.83 (3)
N1—C1—O1—C2	−179.43 (2)	S1′′—C1′′—O1′′—C2′′	−0.48 (3)
C1—O1—C2—C3	−175.01 (2)	S1′′—C1′′—N1′′—C4′′	175.539 (17)
C1—N1—C4—C5	−24.37 (3)	O1′′—C1′′—N1′′—C4′′	−4.68 (3)
C1—N1—C4—C9	159.68 (3)	N1′′—C1′′—O1′′—C2′′	179.75 (2)
S1′—C1′—O1′—C2′	8.49 (2)	C1′′—O1′′—C2′′—C3′′	176.02 (2)
S1′—C1′—N1′—C4′	176.533 (16)	C1′′—N1′′—C4′′—C5′′	36.62 (3)
O1′—C1′—N1′—C4′	−1.49 (3)	C1′′—N1′′—C4′′—C9′′	−147.02 (3)
N1′—C1′—O1′—C2′	−173.540 (19)		

**Table 7:** Hydrogen bond geometry (Å, °) for **2**_NoSpherA2

*D*—H⋯*A*	*D*—H	H⋯*A*	*D*⋯*A*	*D*—H⋯*A*
N1—H1⋯S1^i^	0.996 (5)	2.409 (5)	3.3863 (2)	166.6 (4)
N1′—H1′⋯S1′′	1.014 (5)	2.415 (5)	3.3761 (2)	158.0 (4)
N1′′—H1′′⋯S1′	1.019 (5)	2.371 (5)	3.3668 (2)	165.4 (4)
C5—H5⋯O1	1.075 (4)	2.280 (5)	2.8304 (3)	109.7 (3)
C5′—H5′⋯O1′	1.070 (4)	2.209 (4)	2.8155 (3)	113.7 (3)
C5′′—H5′′⋯O1′′	1.070 (4)	2.319 (4)	2.8002 (3)	105.4 (3)
C9—H9⋯S1^i^	1.072 (4)	2.946 (4)	3.7261 (2)	130.0 (3)
C9—H9⋯S1′^ii^	1.072 (4)	2.922 (5)	3.7860 (3)	137.8 (3)
C9′—H9′⋯S1′′	1.078 (4)	2.844 (5)	3.6073 (3)	127.8 (3)
C9′′—H9′′⋯S1′	1.074 (5)	2.896 (4)	3.6864 (2)	130.6 (3)
C2′—H2′*b*⋯S1^iii^	1.080 (4)	2.886 (4)	3.6869 (3)	131.1 (3)

Symmetry codes: (i) 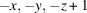; (ii) 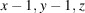; (iii) 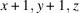.
